# Outcome of proximal femur replacement in failed internal fixation of hip fractures, a case series

**DOI:** 10.1016/j.amsu.2020.04.019

**Published:** 2020-05-12

**Authors:** Zohaib Nawaz, Shah Fahad, Masood Umer, Mujahid Jamil, Younus Durrani, Pervaiz Hashmi

**Affiliations:** Department of Surgery, AKUH, Pakistan

## Abstract

**Introduction:**

Failure of hip implant surgeries can be caused by various factors. Failure of internal fixation results in pain and restricted ambulation. In management of an elderly patient with hip fractures, the aim is to ambulate patient. The purpose of our study is to assess the outcomes of proximal femur replacement in the management of failed hip surgeries for fractures of the proximal femur.

**Materials and methods:**

A retrospective analysis of 26 patients, who underwent proximal femur replacement for failed surgeries of hip fracture during the period from April 2011 to March 2018, was conducted. All patients who underwent proximal femur replacement for failed hip implants were enrolled into the study.

**Results:**

Total patients were 26. The mean follow was (12–91 months). The mean Harris Hip score improved from 26 preoperative to 66.7(45–91). Three patients developed dislocations which were managed with closed reduction. Three patients died within one year of surgery, one patent died of sepsis from implant infection at four months after surgery, one patient died of Myocardial infarction. Three patients developed surgical site infection of which one has superficial surgical site infection which was managed with oral antibiotics, in other case developed deep surgical site infection and was managed with wound debridement and IV antibiotics for 6 weeks, in third wound debridement was done but patient died of sepsis.

**Conclusion:**

Proximal femur replacement with modular stem implant has advantages over conventional hip implant in patients undergoing surgery after failure of internal fixation.

## Highlights

• Failure of hip implant surgeries can be caused by various factors.
• In management of an elderly patient with hip fractures, the aim is to ambulate patient.
• Proximal femur replacement with modular stem implant has advantages over conventional hip implant.

## 1 Introduction

Management of failed hip surgery poses a special challenge to surgeon. Failure can be caused by infection, nonunion, malunion, avascular necrosis of femur head, implant failure, loosening periprosthetic fracture [1]. Failure of internal fixation results in pain and restricted ambulation which is further complicated by deep venous thrombosis and pulmonary embolism [2,3].

Treatment options include repeat internal fixation which gives good results in young patients with good quality of bone. Bone grafting and cement supplementation have been used with satisfactory results as well [4,5]. In management of an elderly patient with hip fractures, the aim is to ambulate patient immediately and to restore the pre-operative ambulation status as soon as possible. This is to prevent systemic complication and improve survival and such conservative treatments are not suitable [3,6]. In patients with limited functional demands Girdlestone procedure can be considered but the results are often seen to be poor [7]. In most cases, for older people, the procedure of choice is hip replacement which generally provides good results [2,6].

Use of cemented total hip arthroplasty in the treatment of failed internal fixation is challenging due to poor bone quality at proximal femur and difficulty in containment of the cement due to preexisting holes of internal fixation [8]. Uncemented hip revision arthroplasty implants are designed to obtain fixation in distal femur and bypass the proximal bone deficient segment. Thus proving that an un cemented hip revision arthroplasty in these cases would appear attractive, as these implants are designed to bypass regions of proximally deficient bone and to obtain stability and fixation in the distal femoral bone where there is good bone stock.

The purpose of our study is to assess the outcomes of proximal femur replacement in the management of failed hip surgeries for fractures of the proximal femur. We studied the early clinical and radiographic results as well as the complications.

## 2 Materials and Methods

This is a single-center, multi-operator study conducted at Aga Khan University Hospital which is tertiary-care level-1 trauma center. We obtained the hospital ethical review committee approval and registered the study in data registry.

A retrospective analysis of 26 patients, who underwent proximal femur replacement for failed surgeries of hip fracture during the period from April 2011 to March 2018, was conducted. All orthopedic patients who underwent proximal femur replacement for failed internal fixation of intertrochanteric fractures, sub trochanteric fracture, failed hemi arthroplasties with ambulatory patient before trauma with complete follow up for one year at least, were enrolled into the study. Patients with missing records and those who were lost to follow-up, nonambulatory patients before injury, patients with pathological fractures, infected cases and patient with associated other fractures were excluded. The decision to perform a hemi arthroplasty or a THA was made based on preoperative functional demands of the patient and intraoperative assessment of the condition of the acetabular articular cartilage. Data collected included: age, gender, comorbidities, type of failed internal fixation and hemi arthroplasty.

Pre-operative assessment included a thorough history, physical examination. Medical co-morbidities were recorded and controllable risk factors identified and optimized before surgery. Radiographic evaluation included radiographs of the pelvis and femur anteroposterior and lateral shoot through. Laboratory investigations included complete blood count, serum urea, creatinine, electrolytes, blood sugar random and urine routine examination. A single dose of low molecular weight heparin was administered 12 hours before the surgery.

All procedures were performed under general anesthesia or spinal anesthesia. Intravenous prophylactic antibiotics were administered at the time of induction of anesthesia as according to our institution's guidelines. All procedures were performed by three fellowship trained consultants and as per surgeon preference through lateral or posterior approach. Preoperatively, templating was done with use of fractured and contralateral hip plain radiographs to measure femoral head size, canal diameter as well as length tip of greater trochanter to lesser trochanter. Careful exposure of the hip was obtained sparing abductor mechanism and already intact grater trochanter. Implant of previous surgery was removed. After removing head and clearing acetabulum, un cemented acetabular component was implanted after proper preparation and attention were then turned to the femur (Fig. 1 a, Fig. 1 b, Fig. 1 c c). Femoral canal preparation was done by using progressive reamer sizes until adequate fit was achieved. Same size stem as last reamer was used to trial with a body size to recreate original metaphyseal component, head and neck sizes were adjusted to achieve ample stability with full functional range of motion, maintenance of tissue tension and limb length equality(Fig. 2 b, Fig. 2 a b). Implant used included standard 170mm cementless HA coated femoral stems as well as HA coated primer body with tendon holes(Fig. 1 a); bipolar head was size 28 or 32 as per cup size, stainless steel head with high molecular weight polyethylene. After implantation of the definitive prosthesis, heavy non-absorbable sutures were used to anchor the great trochanter and abductors to the lateral part of proximal region of the prosthesis in which dedicated holes are present(Fig. 3 a, Fig. 3 bb). If abductors were unavailable to be tagged to the prosthesis, an abduction brace was used for six weeks.

**Fig. 1 a:**
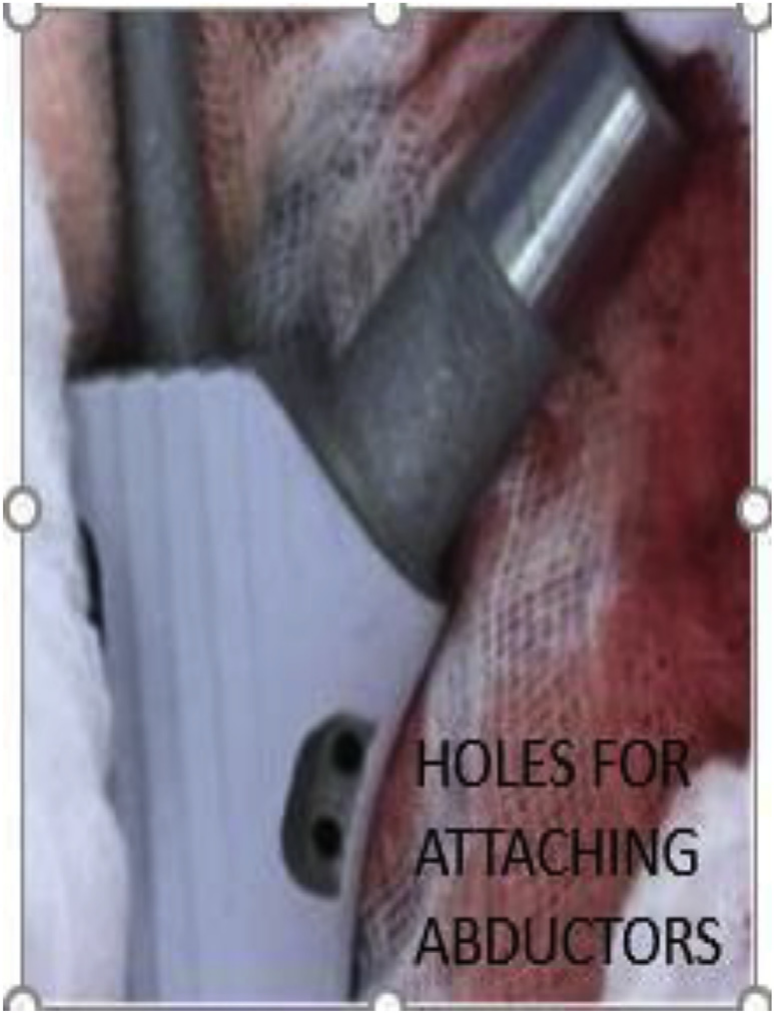
Holes in the proximal femoral component used for securing during trial implantation.

**Fig. 1 b:**
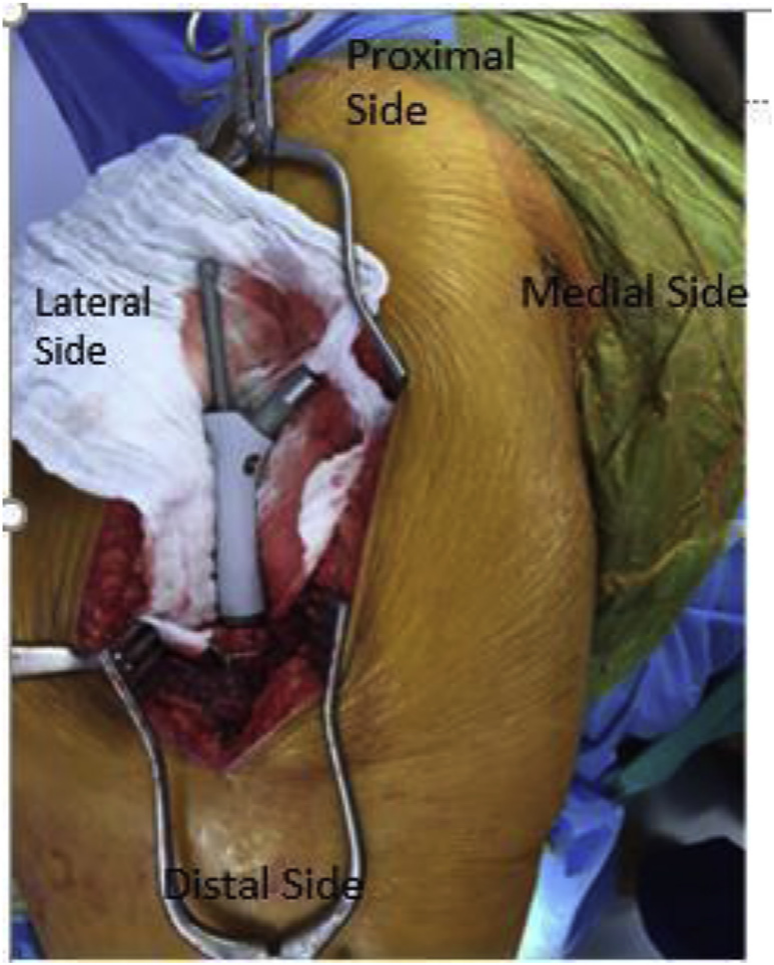
Stay sutures taken iniliopsoas and abductors at the time of trial implantation which are later secured through holes in the proximal femoral component.

**Fig. 1 c:**
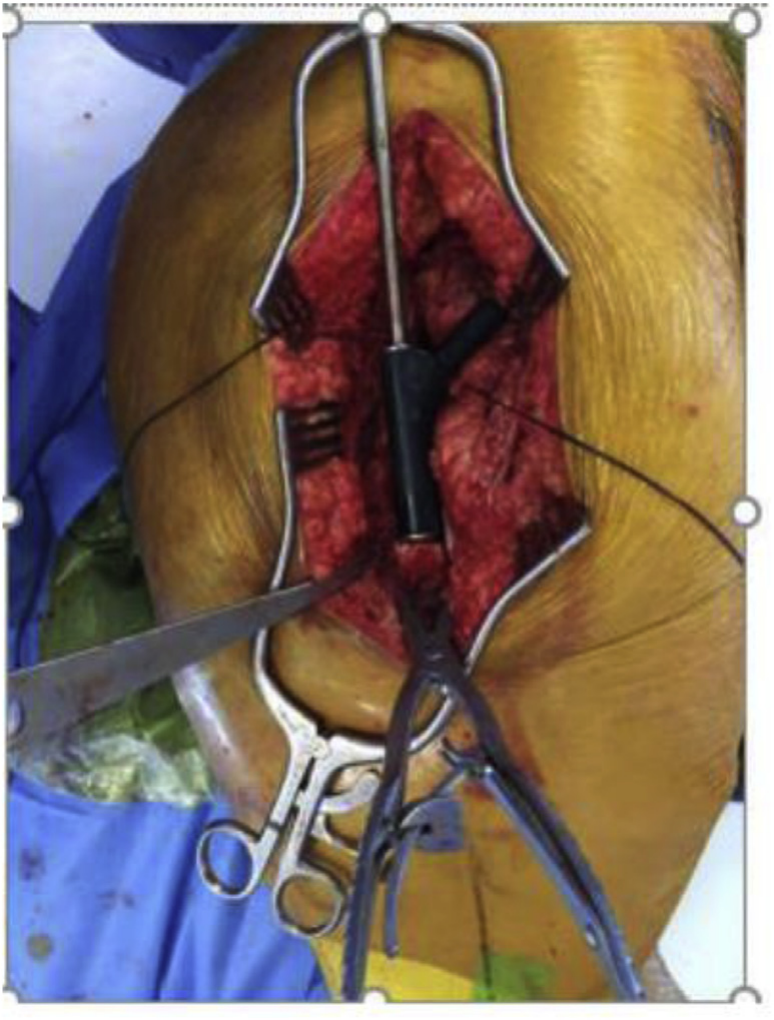
Proximal femoral replacement component along with stem intraoperatively.

**Fig. 2 a:**
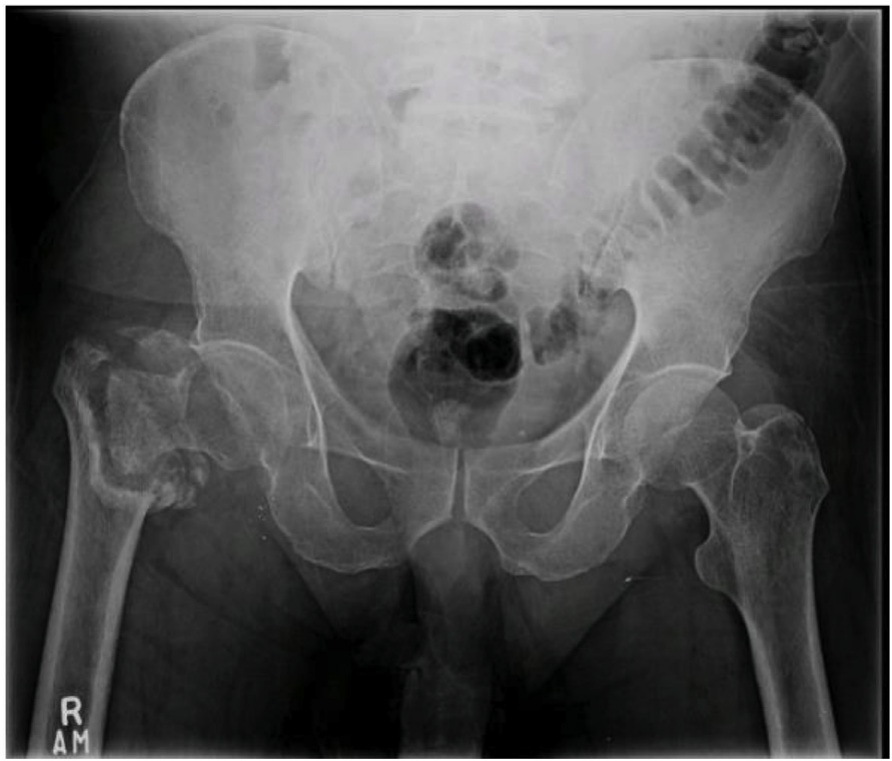
Fracture occurring during removal of previous implant and fixation by dynamic hip screw.

**Fig. 2 b:**
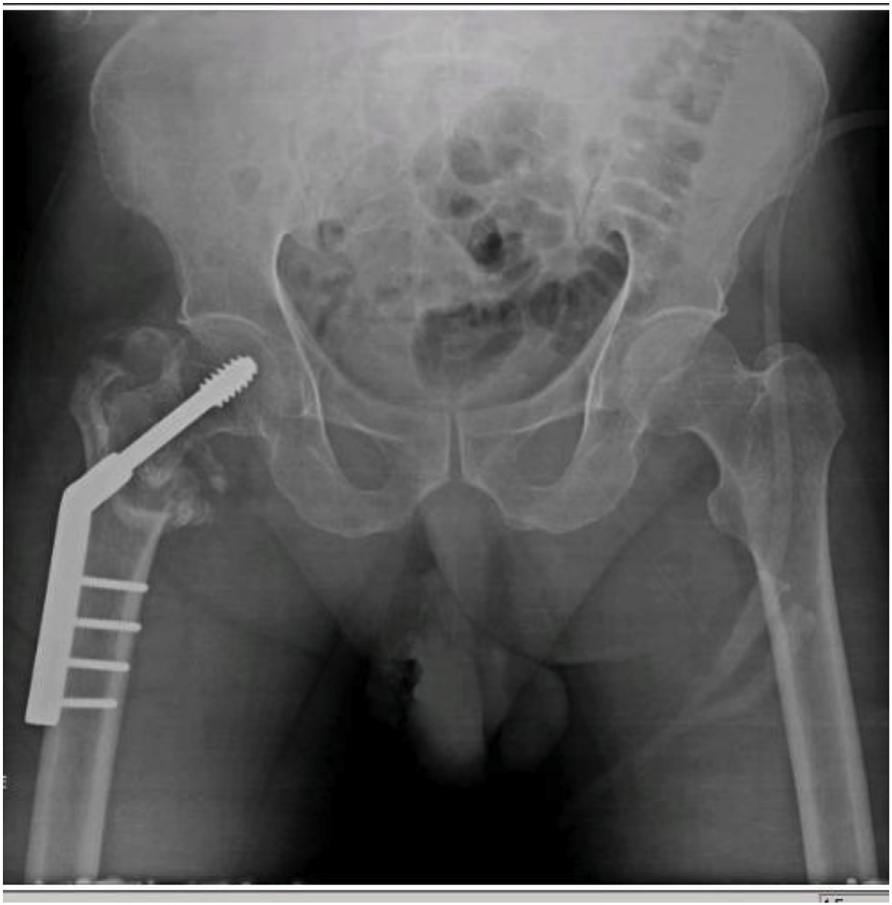
Dynamic hip screw showing non-union of fracture.

**Fig. 3 a:**
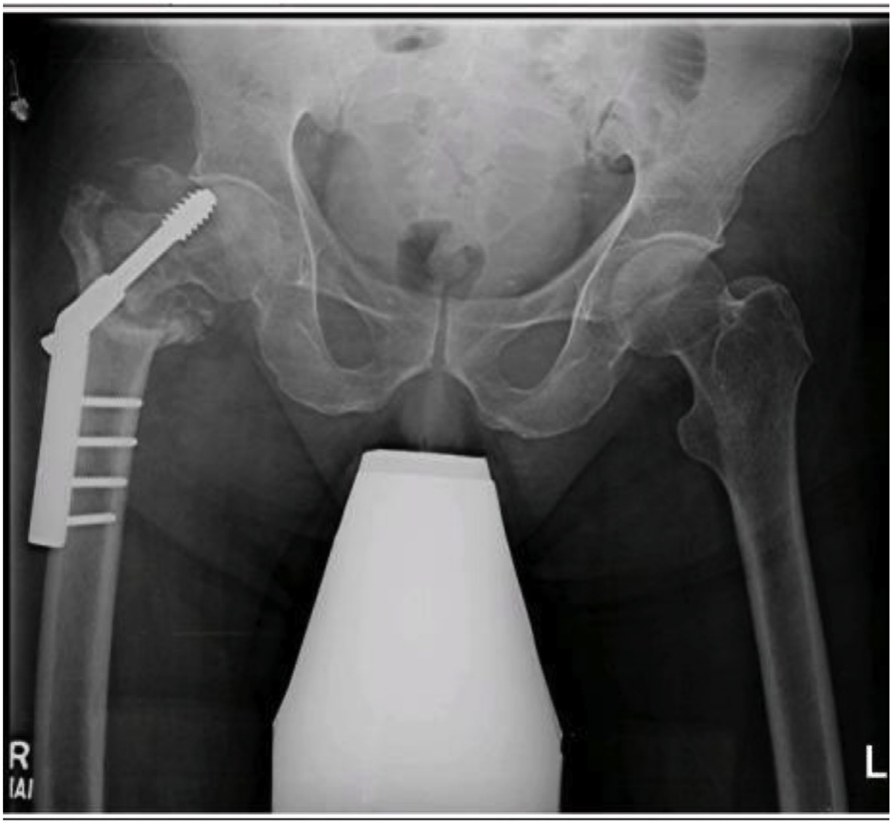
Post dynamic screw insertion, patient presented with non-union of fracture with bone deficiency.

**Fig. 3 b:**
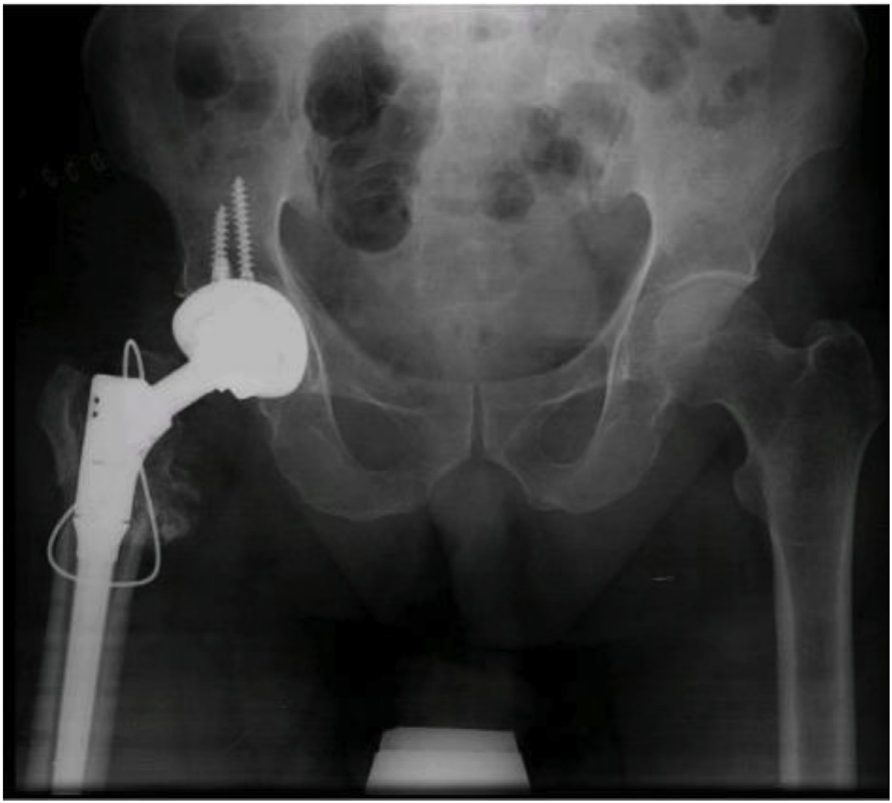
Hip implant with modular stem used to bypass bone deficiency and achieve fixation at the distal and intact part of femur.

Post-operatively, the patients were allowed weight-bearing with ambulation as tolerated with the help of walker. Quadriceps muscle strengthening exercises were started from post op day one. DVT prophylaxis was given as low molecular weight heparin to all patients. Patients were discharged after an average of three days and followed-up one week, two weeks and six monthly during first year after surgery and yearly subsequently. At every visit, the patients were examined clinically for wound healing, postoperative ambulation status, need for walking aid, postoperative complications, one-year mortality. Union time were recorded on every follow-up visit. Radiographs were evaluated for the evidence of loosening. Functional assessment was done via Harris Hip Score. The work has been reported in line with PROCESS criteria [17].

## 3 Results

Total number of patients enrolled were 26, out of which 20 (66.6%) were female and six (33.3%) were male. The mean age of the patients was 74 0.95(range 33–88) years. The mean follow up was (12–91 months).20(76.9%) of the patients were community ambulant with the aid of some support. The mean Harris Hip score improved from 26 preoperative to 66.7(45–91) at last follow up. Post operatively two patients developed acute renal failure which was managed with supportive care. Three patients developed dislocations which were managed with closed reduction. Three patients died within one year of surgery, one patent died of sepsis from implant infection at four months after surgery, one patient died of Myocardial infarction. Three patients developed surgical site infection of which one has superficial surgical site infection which was managed with oral antibiotics, in other case developed deep surgical site infection and was managed with wound debridement and IV antibiotics for 6 weeks, in third wound debridement was done but patient died of sepsis.

## 4 Discussion

Failed internal fixation of proximal femur fracture can be treated with revision internal fixation or hip replacement. Patients with good bone stock and preserved hip joint can be better treated with revision internal fixation plus bone grafting. However, patient with poor bone stock and those with osteoarthritis of hip joint can be better treated with hip replacement [9]. Recent advances in internal fixation implant and surgical technique has improved the outcome of proximal femur fractures, however failures still happen [6].

Proximal femur replacement with modular stem implant has advantages over conventional hip implant in patients undergoing surgery after failure of internal fixation. These implants allow adjustment of leg length, offset and anteversion. It allows separate preparation of the proximal and distal bone in the femur to maximize prosthesis fill [10]. These implants are designed to bypass the region of deficient bone in proximal femur and achieve stability and fixation in the more distal and intact part of femur [11]. The major advantage of this implant is that it can be modulated by the surgeon during surgery according to femur anatomy. The proximal femoral primer body is available in different sizes and the surgeon can choose body of patient sizes to match the femoral metaphysis. In addition, necks of different styles are available which allow the surgeon to modify the offset. The proximal screw allows the rotation of neck and body to control anteversion before tightening. The stems are available in different sizes which provide the opportunity to achieve purchase in distal femur and control limb length [12].

Technical challenges during performing hip arthroplasty for failed proximal femur fractures are the presence of previous implant, and fracture can occur during its removal which can be fixed with dynamic hip screw. This is further compounded by nonunion at proximal femur, bone defect, osteoporosis and holes left after removal of hardware. All these factors encourage fracture of proximal femur during implant insertion and limit proximal femur implant fixation [6]. This can be overcome by calcar replacement implant or implant with longer distal fixation implant [13]. In our case we use a hip implant with modular stem to bypass the bone deficiency and to achieve fixation in the more distal and intact part of the femur.

A retrospective study by Mehlhoff et al. on 27 patients with total hip arthroplasty (THA) following failure of internal fixation of fractures of the proximal femur with the results were less satisfactory. Bone loss and medial displacement of the femoral shaft led to high incidence of intraoperative complications and postoperative dislocations [14]. Tbash et al. observed increased complication and surgical difficulties in Total hip replacement performed for failed fracture treatment [15]. Carmelo et al. conducted a study on 21 patients with failed intertrochanteric fracture treated with total hip replacement with long stem. He observed good results in 47.6% of cases with 9.5% intra and post-operative complication [13], while our post-operative complication rate was 11.5%. In our study we observed significant improvement in post-operative functional status. The mean Harris Hip score improved from 26 preoperative to 66.7(45–91) at last follow up. We encountered 11.5% one-year mortality. This high percentage is partly associated with multiple comorbidities and surgical site infection which these patient have and is comparable with literature [16].

The weaknesses of our study include the retrospective study design and short follow-up. The strength was the use of only 1 design of hip prosthesis. Moreover, in terms of numbers this is the largest analysis to date of an uncemented modular THA for salvage of proximal femoral fracture failure.

## 5 Conclusion

Proximal femur replacement is a viable option in elderly patients undergoing surgery for failed hip surgery, and has been associated with good clinical outcome. Further prospective comparative studies are required to enlightened this topic.

## Source of funding

None.

## Provenance and peer review

Not commissioned, externally peer reviewed.

## Declaration of competing interest

None.
